# Inflammatory and vascular biomarkers as predictors of all‐cause death and cardiovascular outcomes in an Australian community‐based cohort

**DOI:** 10.14814/phy2.70379

**Published:** 2025-06-10

**Authors:** Silvia Lee, Alison Castley, Matthew Knuiman, David Nolan, Frank Sanfilippo, Girish Dwivedi

**Affiliations:** ^1^ Harry Perkins Institute of Medical Research University of Western Australia Murdoch Western Australia Australia; ^2^ Department of Microbiology Pathwest Laboratory Medicine Perth Western Australia Australia; ^3^ Department of Clinical Immunology Pathwest Laboratory Medicine Perth Western Australia Australia; ^4^ School of Population and Global Health University of Western Australia Perth Western Australia Australia; ^5^ Department of Clinical Immunology Royal Perth Hospital Perth Western Australia Australia; ^6^ Department of Cardiology Fiona Stanley Hospital Murdoch Western Australia Australia

**Keywords:** Busselton health survey, cardiovascular disease, CD14, E‐selectin, GDF‐15, outcomes, ST2

## Abstract

Biomarkers that identify individuals who are at higher risk of cardiovascular outcomes will allow for early intervention, lowering the incidence of adverse outcomes. This study investigated whether circulating levels of GDF‐15, E‐selectin, CD14, and ST2 are predictors of death and cardiovascular outcomes in 981 individuals who did not have a history of cardiovascular disease (CVD) during follow‐up periods of 5, 10, and 20 years. During the 20‐year follow‐up, there were 389 deaths (including 147 from CVD), 105 participants had acute coronary syndrome (ACS), and 467 people had major adverse coronary and cerebrovascular events (MACCE) (including all‐cause death). In the fully adjusted model, sE‐selectin (5‐year HR, 3.03; 95% CI 1.31–7.01), sCD14 (5 years 3.11; 1.02–9.45 and 10 years 2.52; 1.23–5.16), and sGDF‐15 (10 years 2.07; 1.13–3.78 and 20 years 1.79; 1.24–2.56) predicted all‐cause death. sE‐selectin (5 years 2.19; 1.13–4.26), sCD14 (10 years 2.00; 1.08–3.68), and sGDF‐15 (10 years 1.95; 1.18–3.22 and 20 years 1.53; 1.11–2.12) predicted MACCE. sGDF‐15 predicted ACS at 5 (4.44; 1.01–19.49), 10 (2.86; 1.08–7.57) and 20 years (2.57; 1.31–5.04). High serum levels of sE‐selectin, sGDF‐15, and sCD14 at baseline are important independent risk factors for all‐cause death and cardiovascular outcomes in a population without prevalent CVD.

## 1 INTRODUCTION

Cardiovascular disease (CVD) is one of the leading health problems in the world, contributing to approximately 17.9 million deaths in 2015. Atherosclerosis is a common cause of CVD and is characterized by the accumulation of lipids in the arterial wall (Xu et al., 2019). This results in narrowing of the arteries, and subsequent plaque rupture can lead to acute coronary syndrome (ACS) and stroke (Lee et al., 2020). Atherosclerosis is now considered a chronic maladaptive inflammatory disease (Bartlett et al., 2019).

Hypertension, hypercholesterolemia, and diabetes mellitus are well‐established independent cardiovascular risk factors, but several biomarkers have now emerged that show added predictive value for adverse cardiovascular outcomes. Biomarkers to predict cardiovascular risk have been extensively studied in at‐risk populations, including patients with diabetes (Liang et al., 2023), chronic kidney disease (Mohebi et al., 2023) and established cardiovascular disease (Desai et al., 2023). While biomarker studies have also been completed in older community‐based populations, many of these studies included individuals with prevalent CVD at baseline (Jia et al., 2023). Studies of the general population without pre‐existing CVD are scarce.

Atherosclerosis is a complex, multifactorial process involving many cell types capable of producing inflammatory proteins (Madaudo et al., 2024). Suppression of tumorigenicity 2 (ST2) is a member of the interleukin‐1 receptor superfamily and circulating levels are associated with cardiac stress, inflammation, or injury (Aleksova et al., 2019). E‐selectin is a cell adhesion molecule and is released following endothelial activation during the development of atherosclerosis (Zhang, 2022). Growth differentiation factor 15 (GDF‐15) is a member of the transforming growth factor‐β superfamily with key roles in cell growth, apoptosis, and activation of inflammatory responses (Li et al., 2022). CD14 is a pattern recognition receptor for lipopolysaccharide and elevated circulating levels are implicated in diseases associated with monocyte/macrophage activation (Sharygin et al., 2023). In this study, we evaluated serum levels of these markers as predictors of all‐cause death and cardiovascular outcomes in a population of older healthy adults with no history of CVD followed for up to 20 years. Individually, these biomarkers have been found to be predictive of adverse cardiovascular outcomes, but no study has evaluated them all together in the general population.

## 2 METHODS

### 2.1 Study participants

The study included a retrospective cohort of 981 individuals aged 55.9–79.8 years who participated in the 1994/95 survey of the Busselton Health Study and had no history of CVD. The survey data were linked to matching records of hospital admissions from all public and private hospitals in Western Australia and death registry data, both of which were obtained from the Western Australian Department of Health. A history of CVD was identified from the linked data of hospital admissions during the 15 years before the survey date. All participants provided written informed consent at the time of the 1994/95 survey, and the study was approved by the Human Research Ethics Committees of the Western Australian Department of Health (2011/60) and the University of Western Australia (RA/4/1/6682).

### 2.2 Baseline variables

Variables available from the clinical survey dataset and considered as confounders in regression models included age, gender, smoking status (never, ex or current), body mass index (BMI), C‐reactive protein (CRP), comorbidities (diabetes, chronic obstructive pulmonary disease (COPD)), lipids (total cholesterol, high‐density lipoprotein (HDL) cholesterol, triglycerides), estimated glomerular filtration rate (eGFR), blood glucose, systolic blood pressure (SBP), and antihypertensive medications. Clinical assessments were completed as part of the 1994/95 survey. Blood pressure was measured using a mercury sphygmomanometer after 5 min rest in a sitting position. Body mass index was defined as weight (kg) divided by height (m) squared. Serum lipids, glucose, CRP, and creatinine were determined from a fasting blood sample at the time of the survey. Details of smoking, alcohol consumption, diabetes, COPD, and use of antihypertensive drugs were obtained from the survey questionnaire.

### 2.3 Soluble biomarkers

Fasting blood samples were obtained from participants during the 1994/95 survey and stored at −70°C after processing. Aliquots were obtained for use in the current study for those who had a sufficient quantity of stored sample remaining.

Levels of ST2, E‐selectin, and GDF‐15 were determined in the serum from 981 participants using the multiplex Human Magnetic Luminex Assay (Catalogue number LXSAHM10, R&D Systems, Minneapolis, MN, USA) according to the manufacturer's instructions. Quantification of proteins were determined using the Luminex 200™ System. Levels of the inflammatory cytokine, CD14, were quantitated in the serum from 981 participants using Quantikine enzyme‐linked immunosorbent assay (ELISA) kits (Catalogue number DC140, R&D Systems, MN, USA) according to the manufacturer's instructions.

### 2.4 Outcomes

Outcome events for the period from baseline survey to 2014 were determined using linked hospital admissions and death data. Primary endpoints included all‐cause death, cardiovascular death, acute coronary syndrome (ACS) and major adverse coronary and cerebrovascular events (MACCE 1: composite of all‐cause death, ACS, stroke and coronary artery revascularisation procedures). A secondary analysis was performed in which all‐cause death in MACCE 1 was replaced with cardiovascular death in the MACCE definition (MACCE 2). Codes from the International Classification of Diseases 9th revision clinical modification (ICD‐9‐CM) were used for events up to 30 June 1999, and the 10th revision Australian Modification (ICD‐10‐AM) was used for events from 1 July 1999 onwards. Cardiovascular events were defined as hospital admissions with a principal discharge diagnosis of coronary heart disease (ICD‐9‐CM 410–414; ICD‐10‐AM I20‐25) or stroke (ICD‐9‐CM 430–437; ICD‐10‐AM I60‐68) or death from CVD (ICD‐9‐CM 390–459; ICD‐10‐AM I00‐99).

### 2.5 Statistical analysis

Descriptive statistics are presented as mean (standard deviation) for quantitative variables and percentage for categorical variables. Associations between biomarkers and outcomes of death and CVD were determined using Cox proportional hazards regression modeling. To investigate whether associations varied over time, separate proportional hazards models were assessed at 5‐, 10‐, and 20‐year follow‐up. Biomarkers were examined as continuous variables, and due to the skewed distribution of serum levels, we used their natural log transform in the regression models. Cox models evaluated the effects of the four biomarkers individually as well as separate models that included all four biomarkers in the same model for prediction of death and cardiovascular outcomes. The estimated hazard ratios (HRs) with 95% confidence interval (CI) were reported after adjustment for baseline confounders. Three hierarchical levels of adjustment were used. Model 1 adjusted for age and sex. Model 2 further adjusted for smoking, BMI, BP treatment, SBP, diabetes, total cholesterol, HDL cholesterol, triglycerides, blood glucose, and CRP. Model 3 further adjusted for COPD, eGFR, and human cytomegalovirus (HCMV) antibody. Statistical analyses were performed using SAS 9.4 (SAS Institute Inc., NC, USA). *p*‐values <0.05 were considered statistically significant.

## 3 RESULTS

### 3.1 Cohort characteristics

The baseline characteristics of the overall study cohort and in individuals with and without MACCE 1 (with all‐cause death) or MACCE 2 (with cardiovascular death) are summarized in Tables 1 and 2. The overall cohort consisted of 981 individuals with a mean age of 66.6 years, and 41% were male. Mean BMI was 26.8 kg/m^2^; 9.6% were smokers, 8.6% had diabetes, and 26.1% of individuals were taking antihypertensive medication. Mean eGFR was 64.0 mL/min; 12.9% had COPD, and 26.1% individuals were receiving BP treatment.

**TABLE 1:** Baseline characteristics of the study cohort (*n* = 981).

Characteristic	Value
Age (yrs)	66.6 ± 6.5
Males	399 (40.7%)
Smoking status Never	559 (57.0%)
Ex	328 (33.4%)
Current	94 (9.6%)
BMI (kg/m^2^)	26.8 ± 4.2
BP treatment	256 (26.1%)
Systolic BP (mmHg)	133 ± 17
Cholesterol (mmol/L)	6.05 ± 1.05
HDL cholesterol (mmol/L)	1.41 ± 0.41
Triglycerides (mmol/L)	1.49 ± 0.83
Glucose (mmol/L)	5.16 ± 1.21
CRP (mg/L)	4.08 ± 12.76
eGFR (mL/min)	64.0 ± 11.0
Diabetes	84 (8.6%)
COPD	127 (12.9%)
Log sE‐selectin (ng/mL)	3.29 ± 0.40
Log sGDF‐15 (pg/mL)	6.65 ± 0.36
Log sST2 (ng/mL)	2.37 ± 0.34
Log sCD14 (ng/mL)	14.3 ± 0.25

*Note*: Values are presented as mean ± standard deviation or *n* (%).

Abbreviations: BMI, body mass index; BP, blood pressure; COPD, chronic obstructive pulmonary disease; CRP, C‐reactive protein; eGFR, estimated glomerular filtration rate; GDF‐15, growth differentiation factor 15; HCMV, human cytomegalovirus; HDL, high density lipoprotein; ST2, suppression of tumorigenicity 2.

**TABLE 2:** Baseline characteristics of the study cohort stratified by MACCE (*n* = 981).

Characteristic	MACCE 1		MACCE 2	
	No (*n* = 514)	Yes (*n* = 467)	No (*n* = 718)	Yes (*n* = 263)
Age (yrs)	63.4 ± 5.2	70.1 ± 6.1	65.2 ± 6.1	70.4 ± 6.3
Males	187 (36.4%)	212 (45.4%)	281 (39.1%)	145 (55.1%)
Smoking status Never	319 (62.1%)	240 (51.4%)	408 (56.8%)	151 (57.4%)
Ex	159 (30.9%)	169 (36.2%)	241 (33.6%)	87 (33.1%)
Current	36 (7.0%)	58 (12.4%)	69 (9.6%)	25 (9.5%)
BMI (kg/m^2^)	26.7 ± 4.2	26.9 ± 4.2	26.7 ± 4.2	27.0 ± 4.0
BP treatment	93 (18.1%)	163 (34.9%)	162 (22.6%)	94 (35.7%)
Systolic BP (mmHg)	130.0 ± 16.5	137.1 ± 17.6	131.5 ± 16.9	138.6 ± 17.6
Cholesterol (mmol/L)	6.0 ± 1.1	6.1 ± 1.0	6.0 ± 1.1	6.2 ± 1.0
HDL cholesterol (mmol/L)	1.4 ± 0.4	1.4 ± 0.4	1.4 ± 0.4	1.3 ± 0.4
Triglycerides (mmol/L)	1.4 ± 0.8	1.6 ± 0.9	1.4 ± 0.8	1.6 ± 0.9
Glucose (mmol/L)	5.0 ± 0.7	5.3 ± 1.5	5.1 ± 0.9	5.5 ± 1.7
CRP (mg/L)	3.7 ± 6.6	4.5 ± 17.4	3.6 ± 6.0	5.3 ± 22.5
eGFR (mL/min)	65.3 ± 10.8	62.6 ± 11.0	64.6 ± 10.7	62.4 ± 11.5
Diabetes	30 (5.8%)	54 (11.6%)	45 (6.3%)	39 (14.8%)
COPD	46 (9.0%)	81 (17.3%)	91 (12.7%)	36 (13.7%)
Log sE‐selectin (ng/mL)	3.27 ± 0.39	3.32 ± 0.41	3.27 ± 0.39	3.35 ± 0.41
Log sGDF‐15 (pg/mL)	6.53 ± 0.33	6.78 ± 0.36	6.60 ± 0.35	6.79 ± 0.37
Log sST2 (ng/mL)	2.33 ± 0.33	2.42 ± 0.34	2.35 ± 0.34	2.43 ± 0.34
Log sCD14 (ng/mL)	14.28 ± 0.25	14.33 ± 0.25	14.29 ± 0.25	14.34 ± 0.24
% ≥Median				
Log sE‐selectin (ng/mL)	243 (47.3%)	248 (53.1%)	345 (48.1%)	146 (55.5%)
Log sGDF‐15 (pg/mL)	185 (36.0%)	306 (65.5%)	322 (44.9%)	169 (64.3%)
Log sST2 (ng/mL)	230 (44.8%)	261 (55.9%)	339 (47.2%)	152 (57.8%)
Log sCD14 (ng/mL)	243 (47.3%)	248 (53.1%)	345 (48.1%)	146 (55.5%)

*Note*: Values are presented as mean ± standard deviation or *n* (%).

Abbreviations: BMI, body mass index; BP, blood pressure; COPD, chronic obstructive pulmonary disease; CRP: C‐reactive protein; eGFR, estimated glomerular filtration rate; GDF‐15, growth differentiation factor 15; HCMV, human cytomegalovirus; HDL, high density lipoprotein; MACCE 1, major adverse cardiovascular and cerebrovascular events (composite of all‐cause death, ACS, stroke and coronary artery revascularisation procedure); MACCE 2, major adverse cardiovascular and cerebrovascular events (composite of cardiovascular death, ACS, stroke and coronary artery revascularisation procedure); ST2, suppression of tumorigenicity 2.

During the 20‐year follow‐up period, there were 389 (39.6%) deaths, including 147 (15.0%) deaths attributable to CVD (Table 3). 105 (10.7%) participants had ACS, and 467 (47.6%) people had MACCE 1 (with all‐cause death) whilst 263 (26.8%) had MACCE 2 (with cardiovascular death).

**TABLE 3:** Outcomes at 5, 10, and 20 years follow‐up.

Event	Value
All‐cause death 5 years	55 (5.6%)
10 years	137 (14.0%)
20 years	389 (39.6%)
CV death 5 years	21 (2.1%)
10 years	49 (5.0%)
20 years	147 (15.0%)
ACS 5 years	23 (2.3%)
10 years	50 (5.1%)
20 years	105 (10.7%)
MACCE 1 5 years	84 (8.6%)
10 years	193 (19.7%)
20 years	467 (47.6%)
MACCE 2 5 years	54 (5.5%)
10 years	117 (11.9%)
20 years	263 (26.8%)

*Note*: Values are presented as *n* (%).

Abbreviations: ACS, acute coronary syndrome; CV, cardiovascular; MACCE 1, major adverse cardiovascular and cerebrovascular events (composite of all‐cause death, ACS, stroke and coronary artery revascularisation procedure); MACCE 2, major adverse cardiovascular and cerebrovascular events (composite of cardiovascular death, ACS, stroke and coronary artery revascularisation procedure).

### 3.2 Individual biomarkers and event risk

Results of the single biomarker models are summarized in Table 4 and Tables S1 and S2. Levels of sE‐selectin were associated with all‐cause death at 5 and 20 years, and with CVD death, ACS, and MACCE (with all‐cause death or cardiovascular death included) outcomes at 5‐, 10‐, and 20‐year follow‐up after adjustment for age and sex (model 1; Table S1). These associations remain evident at all follow‐up time points for CVD death and MACCE 2 (with cardiovascular death) outcomes and for all‐cause death and MACCE 1 (with all‐cause death) at 5 years follow‐up after further adjustment for smoking, BMI, BP treatment, SBP, diabetes, total cholesterol, HDL cholesterol, triglycerides, blood glucose, and CRP (model 2; Table S2) and COPD, eGFR, and HCMV antibody (model 3; Table 4).

**TABLE 4:** Adjusted Cox regression analysis relating individual biomarkers to the risk of all‐cause death and cardiovascular outcomes among the study cohort (Model 3).

	All‐cause death^a^		CV death^a^		ACS^a^		MACCE 1^a^, ^c^		MACCE 2^a^, ^d^	
	HR (95% CI)	*p*‐Value	HR (95% CI)	*p*‐Value	HR (95% CI)	*p*‐Value	HR (95% CI)	*p*‐Value	HR (95% CI)	*p*‐Value
sE‐selectin										
5 years	2.24 (1.06–4.75)	**0.0356**	6.40 (1.75–23.38)	**0.0050**	2.90 (0.87–9.69)	0.0847	2.02 (1.10–3.73)	**0.0239**	2.85 (1.30–6.21)	**0.0086**
10 years	1.28 (0.80–2.05)	0.2959	3.90 (1.70–8.94)	**0.0013**	2.07 (0.92–4.66)	0.0795	1.50 (1.00–2.25)	0.0511	2.52 (1.47–4.32)	**0.0008**
20 years	1.23 (0.92–1.63)	0.1607	2.00 (1.24–3.22)	**0.0043**	1.57 (0.90–2.75)	0.1128	1.26 (0.97–1.63)	0.0862	1.52 (1.07–2.16)	**0.0189**
	Interaction with follow‐up time^b^	0.9293	Interaction with follow‐up time^b^	0.2403	Interaction with follow‐up time^b^	0.2990	Interaction with follow‐up time^b^	0.3802	Interaction with follow‐up time^b^	0.0693
sGDF‐15										
5 years	1.81 (0.74–4.44)	0.1977	1.89 (0.44–7.16)	0.3945	5.22 (1.31–20.9)	**0.0193**	2.21 (1.07–4.57)	**0.0331**	2.37 (0.97–5.76)	0.0581
10 years	2.37 (1.34–4.19)	**0.0032**	2.46 (0.94–6.42)	0.0663	3.35 (1.32–8.52)	**0.0109**	2.30 (1.42–3.72)	**0.0007**	2.25 (1.23–4.13)	**0.0086**
20 years	1.88 (1.33–2.67)	**0.0004**	2.53 (1.41–5.52)	**0.0018**	2.97 (1.55–5.68)	**0.0010**	1.64 (1.20–2.24)	**0.0020**	1.71 (1.13–2.59)	**0.0113**
	Interaction with follow‐up time^b^	0.9291	Interaction with follow‐up time^b^	0.4424	Interaction with follow‐up time^b^	0.0619	Interaction with follow‐up time^b^	0.6473	Interaction with follow‐up time^b^	0.6280
sST2										
5 years	0.46 (0.20–1.06)	0.0694	1.56 (0.37–6.55)	0.5463	1.63 (0.42–6.28)	0.4812	0.66 (0.34–1.28)	0.2156	1.22 (0.52–2.89)	0.6461
10 years	0.96 (0.55–1.66)	0.8771	1.50 (0.58–3.90)	0.4015	1.37 (0.55–3.41)	0.4929	1.04 (0.66–1.65)	0.8540	1.26 (0.70–2.28)	0.4381
20 years	1.26 (0.91–1.74)	0.1696	1.66 (0.97–2.83)	0.0624	1.34 (0.72–2.51)	0.3516	1.18 (0.88–1.58)	0.2823	1.24 (0.84–1.83)	0.2869
	Interaction with follow‐up time^b^	**0.0146**	Interaction with follow‐up time^b^	0.5625	Interaction with follow‐up time^b^	0.3190	Interaction with follow‐up time^b^	0.2070	Interaction with follow‐up time^b^	0.7912
sCD14										
5 years	2.96 (1.03–8.52)	**0.0439**	1.42 (0.27–7.65)	0.6804	0.87 (0.16–4.73)	0.8697	1.92 (0.80–4.57)	0.1433	1.28 (0.43–3.83)	0.6567
10 years	2.84 (1.41–5.73)	**0.0036**	0.86 (0.28–2.63)	0.7901	1.34 (0.42–4.31)	0.6184	2.29 (1.26–4.15)	**0.0066**	1.31 (0.62–2.80)	0.4789
20 years	1.36 (0.89–2.07)	0.1600	1.16 (0.59–2.31)	0.6654	2.18 (0.95–5.02)	0.0666	1.37 (0.92–2.02)	0.1172	1.50 (0.89–2.52)	0.1268
	Interaction with follow‐up time^b^	**0.0245**	Interaction with follow‐up time^b^	0.7615	Interaction with follow‐up time^b^	0.8778	Interaction with follow‐up time^b^	0.1304	Interaction with follow‐up time^b^	0.6267

*Note*: All biomarkers variables are log transformed. *P*‐values in bold are statistically significant (*p* < 0.05).

^a^ Model 3: Adjusted for sex, age, smoking, body mass index, blood pressure treatment, systolic blood pressure, diabetes, cholesterol, high density lipoprotein cholesterol, triglycerides, glucose, C‐reactive protein, chronic obstructive pulmonary disease, estimated glomerular filtration rate, and human cytomegalovirus antibody.

^b^ Test for change in hazard ratio over follow‐up time.

^c^ Composite of all‐cause death, ACS, stroke, and coronary artery revascularisation procedure.

^d^ Composite of cardiovascular death, ACS, stroke, and coronary artery revascularisation procedure.

sGDF‐15 levels were associated with ACS, MACCE 1 (with all‐cause death) and MACCE 2 (with cardiovascular death) at all follow‐up time points after adjustment for age and sex (*p* < 0.0001–0.0241, Table S1), but the associations for all of these outcomes were no longer significant at 5 years follow‐up in model 2 (Table S2). In the fully adjusted model (Table 4), sGDF‐15 was a predictor of ACS and MACCE 1 (with all‐cause death) at all follow‐up time points (*p* = 0.0007 to 0.0331), all‐cause death and MACCE 2 (with cardiovascular death) at 10‐ and 20‐year follow‐up (*p* = 0.0004 to 0.0113), and CVD death at 20 years follow‐up (*p* = 0.0018).

sST2 was not predictive of any outcomes except for CVD death at 20‐year follow‐up following adjustment for age and sex (*p* = 0.0394, Table S1).

sCD14 predicted all‐cause death at 5 years (HR, 2.96; 95% CI 1.03–8.52; *p* = 0.0439) and 10 years (HR, 2.84; 95% CI 1.41–5.73; *p* = 0.0036) follow‐up, and MACCE 1 (with all‐cause death) at 10 years (HR, 2.29; 95% CI 1.26–4.15; *p* = 0.0066) follow‐up in the fully adjusted model (Table 4).

In separate analyses, when individuals were dichotomized according to the median levels of the biomarkers, similar associations with MACCE1 (with all‐cause death) and MACCE 2 (with cardiovascular death) were observed (Table S3).

### 3.3 Biomarkers in combination and risk of outcomes

Independent biomarkers were then assessed in combination as predictors of all‐cause death and MACCE. Results are summarized in Table 5. As seen in the individual biomarker models, sE‐selectin (5 years: HR, 3.03; 95% CI 1.31–7.01; *p* = 0.0098), sGDF‐15 (10 years: HR, 2.07; 95% CI 1.13–3.78; *p* = 0.0179 and 20 years: HR, 1.79; 95% CI 1.24–2.56; *p* = 0.0017) and CD14 (5 years: HR, 3.11; 95% CI 1.02–9.45; *p* = 0.045 and 10 years: HR, 2.52; 95% CI 1.23–5.16; *p* = 0.0118) remained significant predictors of all‐cause death when they were combined in the fully adjusted model.

**TABLE 5:** Adjusted Cox regression analysis relating biomarkers to risk of all‐cause death and cardiovascular outcomes among study cohort, including all biomarkers in the model.

	All‐cause death^a^		CV death^a^		ACS^a^		MACCE 1^a^, ^b^		MACCE 2^a^, ^c^	
	HR (95% CI)	*p*‐Value	HR (95% CI)	*p*‐Value	HR (95% CI)	*p*‐Value	HR (95% CI)	*p*‐Value	HR (95% CI)	*p*‐Value
sE‐selectin										
5 years	3.03 (1.31–7.01)	**0.0098**	7.54 (1.76–32.19)	**0.0064**	2.03 (0.55–7.42)	0.2854	2.19 (1.13–4.26)	**0.0203**	2.64 (1.15–6.08)	**0.0227**
10 years	1.13 (0.68–1.87)	0.6308	3.53 (1.42–8.76)	**0.0066**	1.64 (0.69–3.90)	0.2683	1.34 (0.87–2.07)	0.1785	2.28 (1.28–4.05)	**0.0051**
20 years	1.05 (0.78–1.43)	0.7365	1.59 (0.96–2.64)	0.0723	1.29 (0.71–2.35)	0.3991	1.14 (0.86–1.51)	0.3537	1.40 (0.96–2.03)	0.0775
sGDF‐15										
5 years	1.27 (0.50–3.26)	0.6128	1.08 (0.24–4.90)	0.9208	4.44 (1.01–19.49)	**0.0479**	1.78 (0.84–3.81)	0.1349	1.84 (0.73–4.65)	0.1981
10 years	2.07 (1.13–3.78)	**0.0179**	1.64 (0.59–4.60)	0.3442	2.86 (1.08–7.57)	**0.0345**	1.95 (1.18–3.22)	**0.0092**	1.76 (0.94–3.32)	0.0783
20 years	1.79 (1.24–2.56)	**0.0017**	2.14 (1.17–3.91)	**0.0138**	2.57 (1.31–5.04)	**0.0063**	1.53 (1.11–2.12)	**0.0101**	1.50 (0.98–2.30)	0.0645
sST2										
5 years	0.32 (0.13–0.74)	**0.0080**	0.70 (0.16–3.13)	0.6449	1.02 (0.24–4.32)	0.9831	0.48 (0.24–0.95)	**0.0363**	0.84 (0.34–2.03)	0.6912
10 years	0.82 (0.47–1.45)	0.5001	0.91 (0.35–2.43)	0.8569	1.03 (0.40–2.68)	0.9482	0.88 (0.55–1.41)	0.5815	0.92 (0.50–1.70)	0.7933
20 years	1.16 (0.82–1.63)	0.3955	1.36 (0.78–2.38)	0.2770	1.09 (0.56–2.09)	0.8077	1.08 (0.79–1.47)	0.6312	1.07 (0.71–1.61)	0.7543
sCD14										
5 years	3.11 (1.02–9.45)	**0.0453**	2.00 (0.33–12.24)	0.4558	0.61 (0.11–3.52)	0.5839	1.74 (0.70–4.27)	0.2306	1.10 (0.36–3.43)	0.8647
10 years	2.52 (1.23–5.16)	**0.0118**	0.86 (0.26–2.76)	0.7935	1.12 (0.34–3.69)	0.8512	2.00 (1.08–3.68)	**0.0264**	1.17 (0.64–2.55)	0.6952
20 years	1.22 (0.79–1.87)	0.3696	1.06 (0.53–2.13)	0.8613	1.82 (0.79–4.23)	0.1620	1.27 (0.86–1.89)	0.2297	1.43 (0.84–2.42)	0.1872

*Note*: All biomarkers variables are log transformed. *P*‐values in bold are statistically significant (*p* < 0.05).

^a^ Models include sex, age, smoking, body mass index, blood pressure treatment, systolic blood pressure, diabetes, cholesterol, high density lipoprotein cholesterol, triglycerides, glucose, C‐reactive protein, chronic obstructive pulmonary disease, estimated glomerular filtration rate, and human cytomegalovirus antibody, log sE‐selectin, log sGDF‐15, log sST2, log sCD14.

^b^ Composite of all‐cause death, ACS, stroke, and coronary artery revascularisation procedure.

^c^ Composite of cardiovascular death, ACS, stroke, and coronary artery revascularisation procedure.

Two biomarkers predicted CVD death in the fully adjusted model: sE‐selectin (5 years: HR, 7.54; 95% CI 1.76–32.19; *p* = 0.0064 and 10 years: HR, 3.53; 95% CI 1.42–8.76; *p* = 0.0066) and sGDF‐15 (20 years: HR, 2.14; 95% CI 1.17–3.91; *p* = 0.0138).

sGDF‐15 predicted ACS at all follow‐up timepoints (5 years: HR, 4.44; 95% CI 1.01–19.49; *p* = 0.0479; 10 years: HR, 2.86; 95% CI 1.08–7.57; *p* = 0.0345; 20 years: HR, 2.57; 95% CI 1.31–5.04; *p* = 0.0063).

sE‐selectin (5 years: HR, 2.19; 95% CI 1.13–4.26; *p* = 0.0203), CD14 (10 years: HR, 2.00; 95% CI 1.08–3.68; *p* = 0.0264), and sGDF‐15 (10 years: HR, 1.95; 95% CI 1.18–3.22; *p* = 0.0092 and 20 years: HR, 1.53; 95% CI 1.11–2.12; *p* = 0.0101) remained significant predictors of MACCE 1 (with all‐cause death) when combined in the fully adjusted model (Table 5).

In contrast to the single biomarker model, increased serum sST2 levels predicted lower risk of all‐cause death (HR, 0.32; 95% CI 0.13–0.74; *p* = 0.0080) and MACCE 1 (HR, 0.48; 95% CI 0.24–0.95; *p* = 0.0363) at 5 years, but not at 10 and 20 years of follow‐up when combined with other biomarkers in the fully adjusted model (Table 5).

Stratification of individuals based on median cut‐off values gave similar associations between biomarkers with MACCE1 (with all‐cause death) and MACCE2 (with cardiovascular death) (Table S4).

## 4 DISCUSSION

We evaluated the associations of serum levels of four markers of inflammation and endothelial activation (ST2, E‐selectin, GDF‐15 and CD14) with outcomes of all‐cause death and cardiovascular events in a cohort of older healthy adults with no history of CVD followed for up to 20 years. Our findings revealed that in the study cohort, serum levels of sE‐selectin, sGDF‐15, sST2, and sCD14 are independent predictors of all‐cause death and cardiovascular outcomes. Identification of reliable biomarkers to predict those who are at higher risk of cardiovascular outcomes such as ACS may allow for early intervention and improved risk stratification, thereby reducing the overall incidence of adverse cardiovascular events and death.

Circulating levels of sGDF‐15 have been associated with all‐cause death and cardiovascular events in the general population (Ho et al., 2018; Meessen et al., 2021; Rohatgi et al., 2012). In 3523 participants aged >50 years from the Framingham Heart study, sGDF‐15 was associated with incident atherosclerotic CVD after a median follow‐up of 14.3 years (Ho et al., 2018). In another community‐based elderly cohort of 2001 participants aged 65–84 years followed for a median of 10.6 years, sGDF‐15 was associated with all‐cause and cardiovascular mortality (Meessen et al., 2021). However, these studies included participants with underlying CVD or heart conditions (e.g., angina, myocardial infarction, atrial fibrillation, heart failure) at the time of recruitment. In two studies that examined individuals with no history of pre‐existing CVD at baseline, sGDF‐15 levels were found to be associated with all‐cause and cardiovascular mortality at 7 and 11 years follow‐up (Daniels et al., 2011; Rohatgi et al., 2012). The present study showed that sGDF‐15 is a predictor of all‐cause and cardiovascular death at 20‐year follow‐up.

Our study also identified sGDF‐15 as a significant predictor of ACS and MACCE at 10 and 20‐year follow‐up. These associations remained significant after adjustment for traditional risk factors and other biomarkers. Measurements of sGDF‐15 have recently been shown to be useful in detecting the early stages of atherosclerosis (Kiss et al., 2023). A study of elderly Caucasian individuals aged over 65 years without a history of cerebrovascular, cardiovascular, or peripheral artery disease found a link between GDF‐15 and markers of subclinical atherosclerosis such as coronary artery calcium scores and ankle‐brachial index (Kiss et al., 2023).

Several studies have demonstrated no association between levels of sE‐selectin and increased risk of adverse outcomes in patients with carotid atherosclerosis (Hoke et al., 2015) or coronary heart disease (Malik et al., 2001), whereas elevated sE‐selectin levels predicted higher cardiovascular events in patients with diabetes (Matsumoto et al., 2010). There are scarce data on the predictive value of sE‐selectin in the general population. In our study, elevated levels of sE‐selectin predicted short‐ but not long‐term all‐cause death and MACCE outcomes (with all‐cause death included). Furthermore, sE‐selectin was a predictor of CVD death and MACCE (with cardiovascular death included) at all follow‐up time‐points. This is in contrast to a previous study of 1773 individuals aged 45–59 years followed for an average of 15 years, where sE‐selectin was associated with increased risk of cardiovascular death in participants with prior CVD but not in those without a history of CVD (Patterson et al., 2015). However, the study recruited only younger men from the general population. In the population‐based EPIC‐Heidelberg cohort with a median age of 51 years, sE‐selectin was not associated with risk of myocardial infarction after adjustment for established cardiovascular risk factors (Pletsch‐Borba et al., 2019). Similarly, the present study found sE‐selectin was not a predictor of ACS.

CD14 is primarily expressed on monocytes, which play an important role in the initiation and progression of atherosclerosis, and therefore have gained considerable interest as a potential biomarker for predicting cardiovascular events. Our sCD14 results showing an association with increased risk of all‐cause death are consistent with previously reported findings. In the Framingham Heart study, Ho et al. found sCD14 to be a predictor of all‐cause and CVD‐related death (Ho et al., 2018). Similarly, in the Cardiovascular Health Study of 5888 participants aged over 65 years and followed for up to 20 years, elevated sCD14 levels predicted increased risk of all‐cause death, in addition to myocardial infarction, coronary heart disease, and stroke (Reiner et al., 2013). However, our study reported no association between sCD14 and ACS.

Although not significant as an individual biomarker, after adjusting for the other biomarkers, sST2 levels were shown to be significantly associated with a lower risk of all‐cause death and MACCE outcome at 5 years, but not at longer follow‐up periods (10 or 20 years). The lack of associations between sST2 as an individual marker and clinical outcomes may be due to the correlations observed between sST2 with other biomarkers (sGDF‐15 and sE‐selectin) included in the model. This contrasts with previous studies where sST2 levels were found to be independently associated with increased risk of all‐cause and cardiovascular death in the general population (Chen et al., 2013; Wang et al., 2012). However, in the population‐based FINRISK97 study of 8444 healthy participants aged between 25 and 74 years, Hughes et al. found that sST2 did not predict CVD events, heart failure, MACE, and all‐cause death (Hughes et al., 2014). Discrepancies between studies could arise due to the different assays used to measure sST2 levels. Previous studies used the FDA approved Presage ST2 assay compared to the R&D Systems Luminex assay used in the present study. However, using the Luminex assay, but with modified beads from Radix Biosolutions, Chen et al. found high sST2 levels to be independently associated with increased risk of all‐cause death in the Dallas Heart study, a population‐based cohort of 3294 participants followed for a median of 8.3 years (Chen et al., 2013). The study differed from ours in that the cohort was younger (30–65 years), approximately half of the participants were African Americans, and sST2 was detectable in only 33% of participants. It is well known that sST2 functions as a decoy receptor for IL‐33, a cytokine known to have cardioprotective effects and limits atherosclerotic plaque progression (Thanikachalam et al., 2023). However, several in vitro studies have demonstrated protective effects of sST2 by down‐regulating production of pro‐inflammatory cytokines following lipopolysaccharide stimulation (Sweet et al., 2001; Takezako et al., 2006). The significance of our results requires further validation, and concomitant measurement of IL‐33 is warranted as the sST2/IL‐33 ratio in the circulation may be a better indicator of the biological functions of IL‐33, and hence of sST2.

Our study has several limitations that have to be taken into account when interpreting the results. Firstly, the study involved retrospective analyses and therefore it is difficult to ascertain the cause‐and‐effect relationship. Longitudinal biomarker measurements were not available, and we could not assess the predictive value of the change in biomarker levels over time. A recent study found that the change in sST2 levels measured for a median of 2.5 years apart (but not baseline levels) was a predictor of incident MACE in an asymptomatic community‐based population (Watson et al., 2020). However, in another study, repeated sGDF‐15 measurements only marginally improved risk prediction for death from coronary heart disease in the general population when compared to a single measurement (Fluschnik et al., 2018). Due to the overall better health status of the participants at baseline, we observed a low frequency of events and therefore the statistical power to detect associations may have been limited. Whilst we have adjusted for traditional cardiovascular risk factors, we cannot exclude the possible effects from residual confounders. Finally, our study included a predominantly Caucasian population and therefore our results need to be confirmed in other non‐Caucasian populations.

In conclusion, baseline levels of sE‐selectin, sGDF‐15, and sCD14 in serum are important independent risk factors for all‐cause death and cardiovascular outcomes in a population without prevalent CVD, whilst sST2 is predominantly protective. Inclusion of these biomarkers in future risk prediction models will allow improved guidance on clinical management decisions to prevent adverse cardiovascular events.

## FUNDING INFORMATION

This work was supported by a Vanguard grant from the National Heart Foundation (Australia, ID 102181) and a Royal Perth Hospital Medical Research Foundation grant (ID 2018/19).

## CONFLICT OF INTEREST STATEMENT

The authors declare no conflict of interest.

## ETHICS STATEMENT

The study was approved by the Human Research Ethics Committees of the Western Australian Department of Health (2011/60) and the University of Western Australia (RA/4/1/6682). All participants provided written informed consent at the time of the 1994/95 survey.

## Supporting information


Table S1.
