# Supplementary material for: Inflammatory and vascular biomarkers as predictors of all‐cause death and cardiovascular outcomes in an Australian community‐based cohort
Source: Physiol Rep. 2025 Jun 10;13(11):e70379. doi: 10.14814/phy2.70379 (PMC12151874; doi:10.14814/phy2.70379)
Supplement: Supplementary file 1 — Table S1. [file PHY2-13-e70379-s001.docx]

**Supplementary Table 1. Adjusted Cox regression analysis relating individual biomarkers to risk of all-cause death and cardiovascular outcomes among the study cohort (Model 1).**

|  | **All-cause death^1^** | | **CV death^1^** | | **ACS^1^** | | **MACCE 1^1, 2^** | | **MACCE 2^1, 3^** | |
| --- | --- | --- | --- | --- | --- | --- | --- | --- | --- | --- |
|  | HR (95% CI) | p-value | HR (95% CI) | p-value | HR (95% CI) | p-value | HR (95% CI) | p-value | HR (95% CI) | p-value |
| **sE-selectin** |  |  |  |  |  |  |  |  |  |  |
| 5 years | 2.22 (1.14-4.33) | **0.0197** | 5.42 (1.87-15.75) | **0.0019** | 4.32 (1.53-12.20) | **0.0058** | 2.29 (1.31-3.99) | **0.0036** | 3.60 (1.81-7.18) | **0.0003** |
| 10 years | 1.43 (0.93-2.19) | 0.1013 | 3.84 (1.91-7.71) | **0.0002** | 2.40 (1.16-4.97) | **0.0181** | 1.73 (1.19-2.51) | **0.0039** | 3.03 (1.88-4.88) | **<0.0001** |
| 20 years | 1.49 (1.14-1.94) | **0.0032** | 2.25 (1.47-3.47) | **0.0002** | 1.84 (1.11-3.07) | **0.0192** | 1.52 (1.19-1.93) | **0.0008** | 1.88 (1.36-2.60) | **0.0001** |
|  |  |  |  |  |  |  |  |  |  |  |
| **sGDF-15** |  |  |  |  |  |  |  |  |  |  |
| 5 years | 1.80 (0.78-4.13) | 0.1659 | 1.93 (0.51-7.35) | 0.3355 | 5.70 (1.62-20.12) | **0.0068** | 2.19 (1.11-4.33) | **0.0241** | 2.66 (1.15-6.17) | **0.0226** |
| 10 years | 2.53 (1.49-4.31) | **0.0006** | 2.07 (0.86-4.98) | 0.1034 | 3.61 (1.47-8.85) | **0.0050** | 2.48 (1.57-3.92) | **<0.0001** | 2.34 (1.31-4.20) | **0.0043** |
| 20 years | 2.19 (1.58-3.04) | **<0.0001** | 2.50 (1.47-4.24) | **0.0007** | 3.40 (1.81-6.39) | **0.0001** | 1.89 (1.40-2.56) | **<0.0001** | 1.91 (1.28-2.84) | **0.0014** |
|  |  |  |  |  |  |  |  |  |  |  |
| **sST2** |  |  |  |  |  |  |  |  |  |  |
| 5 years | 0.54 (0.24-1.22) | 0.1374 | 1.84 (0.47-7.17) | 0.3798 | 1.69 (0.45-6.34) | 0.4334 | 0.75 (0.39-1.47) | 0.4047 | 1.42 (0.61-3.32) | 0.4157 |
| 10 years | 0.99 (0.59-1.67) | 0.9685 | 1.73 (0.71-4.22) | 0.2293 | 1.44 (0.60-3.49) | 0.4160 | 1.11 (0.71-1.74) | 0.6377 | 1.43 (0.80-2.54) | 0.2274 |
| 20 years | 1.28 (0.94-1.75) | 0.1135 | 1.70 (1.03-2.81) | **0.0394** | 1.42 (0.77-2.61) | 0.2576 | 1.20 (0.90-1.59) | 0.2117 | 1.30 (0.89-1.89) | 0.1775 |
|  |  |  |  |  |  |  |  |  |  |  |
| **sCD14** |  |  |  |  |  |  |  |  |  |  |
| 5 years | 3.29 (1.12-9.62) | **0.0303** | 2.03 (0.36-11.33) | 0.4217 | 1.06 (0.21-5.40) | 0.9410 | 2.14 (0.90-5.12) | 0.0867 | 1.59 (0.54-4.69) | 0.3989 |
| 10 years | 3.08 (1.54-6.14) | **0.0014** | 0.98 (0.32-3.00) | 0.9653 | 1.47 (0.47-4.56) | 0.5099 | 2.46 (1.37-4.42) | **0.0025** | 1.41 (0.67-2.98) | 0.3636 |
| 20 years | 1.40 (0.93-2.10) | 0.1091 | 1.31 (0.67-2.54) | 0.4277 | 2.16 (0.97-4.78) | 0.0589 | 1.40 (0.96-2.04) | 0.0825 | 1.57 (0.95-2.60) | 0.0791 |

^1^ Model 1: Adjusted for sex and age.

^2^ composite of all-cause death, acute coronary syndrome, stroke and coronary artery revascularisation procedure

^3^ composite of cardiovascular death, acute coronary syndrome, stroke and coronary artery revascularisation procedure

All biomarkers variables are log transformed.

**Supplementary Table 2. Adjusted Cox regression analysis relating individual biomarkers to risk of all-cause death and cardiovascular outcomes among the study cohort (Model 2).**

|  | **All-cause death^1^** | | **CV death^1^** | | **ACS^1^** | | **MACCE 1^1, 3^** | | **MACCE 2^1, 4^** | |
| --- | --- | --- | --- | --- | --- | --- | --- | --- | --- | --- |
|  | HR (95% CI) | p-value | HR (95% CI) | p-value | HR (95% CI) | p-value | HR (95% CI) | p-value | HR (95% CI) | p-value |
| **sE-selectin** |  |  |  |  |  |  |  |  |  |  |
| 5 years | 2.29 (1.09-4.82) | **0.0294** | 6.21 (1.75-21.97) | **0.0047** | 3.10 (0.92-10.48) | 0.0681 | 2.04 (1.11-1.02) | **0.0216** | 2.84 (1.31-6.17) | **0.0083** |
| 10 years | 1.32 (0.83-2.10) | 0.2439 | 4.24 (1.85-9.74) | **0.0007** | 2.04 (0.91-4.59) | 0.0848 | 1.50 (1.00-2.24) | 0.0518 | 2.45 (1.44-4.19) | **0.0010** |
| 20 years | 1.23 (0.92-1.63) | 0.1609 | 2.03 (1.27-3.26) | **0.0033** | 1.54 (0.88-2.68) | 0.1305 | 1.25 (0.96-1.62) | 0.0933 | 1.50 (1.06-2.13) | **0.0217** |
|  | Interaction with follow-up time^2^ | 0.9695 | Interaction with follow-up time^2^ | 0.2376 | Interaction with follow-up time^2^ | 0.2906 | Interaction with follow-up time^2^ | 0.3530 | Interaction with follow-up time^2^ | 0.0671 |
| **sGDF-15** |  |  |  |  |  |  |  |  |  |  |
| 5 years | 1.70 (0.72-4.05) | 0.2272 | 1.73 (0.43-7.01) | 0.4426 | 3.79 (0.96-14.91) | 0.0566 | 1.89 (0.94-3.82) | 0.0761 | 1.92 (0.81-4.57) | 0.1403 |
| 10 years | 2.15 (1.24-3.74) | **0.0064** | 1.94 (0.77-4.93) | 0.1609 | 2.81 (1.11-7.07) | **0.0285** | 2.03 (1.28-3.25) | **0.0029** | 1.85 (1.02-3.34) | **0.0432** |
| 20 years | 1.79 (1.28-2.50) | **0.0006** | 2.32 (1.34-4.04) | **0.0028** | 2.74 (1.44-5.21) | **0.0021** | 1.56 (1.15-2.11) | **0.0039** | 1.60 (1.07-2.38) | **0.0226** |
|  | Interaction with follow-up time^2^ | 0.9652 | Interaction with follow-up time^2^ | 0.5061 | Interaction with follow-up time^2^ | 0.0684 | Interaction with follow-up time^2^ | 0.6061 | Interaction with follow-up time^2^ | 0.6365 |
| **sST2** |  |  |  |  |  |  |  |  |  |  |
| 5 years | 0.47 (0.21-1.07) | 0.0720 | 1.55 (0.37-6.46) | 0.5506 | 1.59 (0.40-6.25) | 0.5092 | 0.66 (0.34-1.28) | 0.2192 | 1.22 (0.51-2.90) | 0.6586 |
| 10 years | 0.93 (0.54-1.60) | 0.8040 | 1.50 (0.58-3.84) | 0.4027 | 1.34 (0.54-3.32) | 0.5309 | 1.03 (0.65-1.62) | 0.9011 | 1.25 (0.69-2.26) | 0.4557 |
| 20 years | 1.21 (0.88-1.67) | 0.2435 | 1.62 (0.96-2.74) | 0.0723 | 1.33 (0.71-2.48) | 0.3687 | 1.15 (0.86-1.53) | 0.3634 | 1.23 (0.83-1.81) | 0.3046 |
|  | Interaction with follow-up time^2^ | **0.0207** | Interaction with follow-up time^2^ | 0.6109 | Interaction with follow-up time^2^ | 0.3293 | Interaction with follow-up time^2^ | 0.2451 | Interaction with follow-up time^2^ | 0.7872 |
| **sCD14** |  |  |  |  |  |  |  |  |  |  |
| 5 years | 2.93 (1.02-8.44) | **0.0467** | 1.40 (0.26-7.50) | 0.6940 | 0.88 (0.16-4.80) | 0.8788 | 1.93 (0.80-4.61) | 0.1415 | 1.27 (0.42-3.80) | 0.6706 |
| 10 years | 2.69 (1.34-5.42) | **0.0056** | 0.80 (0.26-2.41) | 0.6902 | 1.33 (0.41-4.27) | 0.6373 | 2.25 (1.24-4.10) | **0.0078** | 1.28 (0.60-2.73) | 0.5290 |
| 20 years | 1.28 (0.84-1.95) | 0.2483 | 1.12 (0.57-2.20) | 0.7383 | 2.17 (0.94-4.99) | 0.0685 | 1.34 (0.90-1.97) | 0.1465 | 1.48 (0.88-2.49) | 0.1364 |
|  | Interaction with follow-up time^2^ | **0.0243** | Interaction with follow-up time^2^ | 0.7587 | Interaction with follow-up time^2^ | 0.8801 | Interaction with follow-up time^2^ | 0.1252 | Interaction with follow-up time^2^ | 0.6290 |

^1^ Model 2: Adjusted for sex, age, smoking, blood pressure treatment, systolic blood pressure, diabetes, cholesterol, high density lipoprotein cholesterol, triglycerides, glucose, C-reactive protein,

^2^ Test for change in hazard ratio over follow-up time

^3^ composite of all-cause death, ACS, stroke and coronary artery revascularisation procedure

^4^ composite of cardiovascular death, ACS, stroke and coronary artery revascularisation procedure

All biomarkers variables are log transformed.

**Supplementary Table 3. Adjusted Cox regression analysis relating individual biomarkers (binary, ref: below median) to risk of MACCE outcomes among the study cohort (Model 3).**

| **Binary measures** | **MACCE 1^1, 3^** | | **MACCE 2^1, 4^** |  |
| --- | --- | --- | --- | --- |
|  | HR (95% CI) | p-value | HR (95% CI) | p-value |
| **sE-selectin** |  |  |  |  |
| 5 years | 1.63 (1.02, 2.60) | **0.0395** | 1.44 (0.80, 2.59) | 0.2240 |
| 10 years | 1.21 (0.89, 1.63) | 0.2228 | 1.48 (1.00, 2.20) | 0.0527 |
| 20 years | 1.13 (0.93, 1.37) | 0.2331 | 1.21 (0.93, 1.56) | 0.1593 |
|  | Interaction with follow-up time^2^ | 0.4535 | Interaction with follow-up time^2^ | 0.3695 |
| **sGDF-15** |  |  |  |  |
| 5 years | 1.35 (0.81, 2.26) | 0.2497 | 1.31 (0.69, 2.47) | 0.4147 |
| 10 years | 1.72 (1.22, 2.43) | **0.0022** | 1.55 (1.00, 2.40) | **0.0488** |
| 20 years | 1.36 (1.10, 1.68) | **0.0047** | 1.27 (0.96, 1.68) | 0.0959 |
|  | Interaction with follow-up time^2^ | 0.8721 | Interaction with follow-up time^2^ | 0.8290 |
| **sST2** |  |  |  |  |
| 5 years | 0.80 (0.50, 1.27) | 0.3436 | 1.24 (0.68, 2.27) | 0.4795 |
| 10 years | 0.98 (0.72, 1.34) | 0.9021 | 1.19 (0.80, 1.78) | 0.3986 |
| 20 years | 1.03 (0.84, 1.24) | 0.8052 | 1.09 (0.84, 1.42) | 0.5077 |
|  | Interaction with follow-up time^2^ | 0.7303 | Interaction with follow-up time^2^ | 0.4064 |
| **sCD14** |  |  |  |  |
| 5 years | 1.28 (0.82, 1.99) | 0.2864 | 0.99 (0.56, 1.72) | 0.9563 |
| 10 years | 1.42 (1.05, 1.90) | **0.0214** | 1.15 (0.79, 1.68) | 0.4659 |
| 20 years | 1.05 (0.87, 1.27) | 0.5914 | 1.15 (0.89, 1.47) | 0.2816 |
|  | Interaction with follow-up time^2^ | 0.0597 | Interaction with follow-up time^2^ | 0.8902 |

^1^ Model 3: Adjusted for sex, age, smoking, body mass index, blood pressure treatment, systolic blood pressure, diabetes, cholesterol, high density lipoprotein cholesterol, triglycerides, glucose, C-reactive protein, chronic obstructive pulmonary disease, estimated glomerular filtration rate and human cytomegalovirus antibody

^2^ Test for change in hazard ratio over follow-up time

^3^ composite of all-cause death, ACS, stroke and coronary artery revascularisation procedure

^4^ composite of cardiovascular death, ACS, stroke and coronary artery revascularisation procedure

All biomarkers variables are log transformed.

**Supplementary Table 4. Adjusted Cox regression analysis relating biomarkers (binary, ref: below median) to risk of MACCE outcomes among study cohort, including all biomarkers in the model.**

| **Binary measures** | **MACCE 1^1, 2^** |  | **MACCE 2^1, 3^** |  |
| --- | --- | --- | --- | --- |
|  | HR (95% CI) | p-value | HR (95% CI) | p-value |
| **sE-selectin** |  |  |  |  |
| 5 years | 1.68 (1.05, 2.69) | **0.0315** | 1.40 (0.77, 2.54) | 0.2658 |
| 10 years | 1.18 (0.87, 1.60) | 0.2875 | 1.43 (0.96, 2.13) | 0.0788 |
| 20 years | 1.12 (0.92, 1.37) | 0.2613 | 1.19 (0.91, 1.55) | 0.2012 |
|  |  |  |  |  |
| **sGDF-15** |  |  |  |  |
| 5 years | 1.33 (0.79, 2.24) | 0.2803 | 1.28 (0.67, 2.44) | 0.4517 |
| 10 years | 1.65 (1.16, 2.34) | **0.0053** | 1.49 (0.96, 2.32) | 0.0753 |
| 20 years | 1.35 (1.09, 1.67) | **0.0056** | 1.24 (0.94, 1.65) | 0.1310 |
|  |  |  |  |  |
| **sST2** |  |  |  |  |
| 5 years | 0.73 (0.46, 1.18) | 0.1972 | 1.15 (0.62, 2.13) | 0.6474 |
| 10 years | 0.93 (0.68, 1.27) | 0.6520 | 1.10 (0.73, 1.65) | 0.6453 |
| 20 years | 0.98 (0.80, 1.19) | 0.8347 | 1.05 (0.81, 1.37) | 0.7244 |
|  |  |  |  |  |
| **sCD14** |  |  |  |  |
| 5 years | 1.20 (0.76, 1.88) | 0.4357 | 0.97 (0.55, 1.69) | 0.9004 |
| 10 years | 1.34 (0.99, 1.80) | 0.0563 | 1.11 (0.76, 1.62) | 0.5913 |
| 20 years | 1.03 (0.85, 1.24) | 0.7624 | 1.13 (0.88, 1.45) | 0.3361 |

^1^ Models include sex, age, smoking, body mass index, blood pressure treatment, systolic blood pressure, diabetes, cholesterol, high density lipoprotein cholesterol, triglycerides, glucose, C-reactive protein, chronic obstructive pulmonary disease, estimated glomerular filtration rate and human cytomegalovirus antibody, log sE-selectin, log sGDF-15, log sST2, log sCD14.

^2^ composite of all-cause death, ACS, stroke and coronary artery revascularisation procedure

^3^ composite of cardiovascular death, ACS, stroke and coronary artery revascularisation procedure

All biomarkers variables are log transformed.
